# Postoperative negative-pressure incision therapy following open colorectal surgery (Poniy): study protocol for a randomized controlled trial

**DOI:** 10.1186/s13063-015-0995-4

**Published:** 2015-10-19

**Authors:** André L. Mihaljevic, Rebekka Schirren, Tara C. Müller, Victoria Kehl, Helmut Friess, Jörg Kleeff

**Affiliations:** Department of Surgery, Klinikum rechts der Isar, Technische Universität München and CHIR-Net Munich, Ismaningerstrasse 22, 81675 Munich, Germany; Institute for Medical Statistics and Epidemiology, Klinikum rechts der Isar, Technische Universtität München, Ismaningerstrasse 22, 81675 Munich, Germany; Current affiliation: The Royal Liverpool and Broadgreen University Hospitals NHS Trust, Prescot Street, Liverpool L7 8XP, UK; Department of General-, Visceral- and Pediatric Surgery, University Hospital Düsseldorf, Heinrich Heine University, Moorenstr. 5, 40225 Düsseldorf, Germany

**Keywords:** Abdominal dressing, Colorectal surgery, Negative-pressure wound therapy, Randomized trial, Surgical site infection, Wound edge protector, Wound infection

## Abstract

**Background:**

Postoperative surgical site infections cause substantial morbidity, prolonged hospitalization, costs and even mortality, and remain one of the most frequent surgical complications. In prospective trials with adequate follow-up, more than 20 % of patients undergoing elective colorectal surgery are affected and methods to reduce surgical site infections are urgently needed. Negative-pressure incision therapy is a novel intervention that holds promise to reduce postoperative wound infection rates, but has not yet been rigorously tested in a randomized controlled trial.

**Methods/Design:**

The aim is to investigate whether the postoperative application of a negative-pressure incision therapy device for 5–7 days reduces the rate of surgical site infections following open elective colorectal surgery by 50 %. This is a randomized, controlled, observer-blinded multicentre clinical trial with two parallel study groups. The primary outcome measure will be the rate of surgical site infections within 30 days postoperatively. Surgical site infections are defined according to criteria of the US Centers for Disease Control and Prevention. Statistical analysis of the primary endpoint measure will be based on the intention-to-treat population. The global level of significance is set at 5 % (two-sided) and the sample size (*n* = 170 per group) is determined to assure a power of 80 %.

**Discussion:**

The Poniy trial will explore whether the rate of surgical site infections can be reduced by the application of a negative-pressure incision therapy device in patients undergoing open elective colorectal surgery. Its pragmatic design guarantees high external validity and clinical relevance.

**Trial registration:**

Deutsches Register Klinischer Studien DRKS00006199.

## Background

### Rationale

Postoperative surgical site infections are one of the most frequent surgical complications and a major cause of postoperative morbidity, prolongation of hospital stay, health care costs and even mortality. An estimated 300,000–500,000 surgical site infections occur in the USA annually [1–4]. In Germany, approximately 60,000–200,000 surgical site infections following surgical interventions are reported every year [5–8].

Despite the implementation of such preventive measures as preoperative antibiotic prophylaxis [9–11] and antiseptic skin cleansing [9, 12], surgical site infection rates in prospective trials using the standardized criteria of the US Centers for Disease Control and Prevention (CDC) remain above 15 % after general abdominal surgery [13–16]. In patients undergoing colorectal surgery, surgical site infection rates as high as 20 % and up to 32 % are reported from randomized controlled trials [14, 15, 17, 18]. National data from the USA support these numbers [19].

Several studies have shown an increase in the mean length of hospital stay by 6 to 24 days if surgical site infections occur [4, 20–23]. The resulting direct costs have to be added to indirect costs such as loss of workforce or insurance payments resulting in substantial expenses for the health care system [24–26].

The most frequent pathogens causing postoperative surgical site infection in colorectal surgery are endogenous pathogens from the patient’s gastrointestinal tract [7, 9]. A significant number of surgical site infections occur after postoperative day 5 [14] and it is was proposed that clearing contaminated secretions from the wound might reduce the incidence of surgical site infection [27]. Negative-pressure incision therapy (NPIT) devices were designed to remove fluids from the incisional wound in the early postoperative phase, to reduce tensile forces across the incision and to protect the incision from external contamination. While positive results have been shown *in vitro* and in animal models, the benefit of NPIT devices in clinical practice remains largely unclear.

### Previous trials

Several previous trials have investigated the effect of NPIT in a variety of settings; however, all trials exhibit considerable risk of bias. Stannard *et al.* [28] compared an NPIT system against standard dressings in a single-centre randomized controlled trial in patients with haematoma or fractures following high-energy trauma. In 44 patients with traumatic haematomas, the duration of wound drainage as well as the rate of surgical site infection was reduced using the NPIT system (surgical site infection rates were reduced by 50 % from 16 % to 8 %). The same authors reported a randomized single-centre study in 117 patients with open traumatic fractures randomized to irrigation and debridement followed by standard fine mesh gauze dressing compared with the same procedure followed by NPIT [29]. The authors reported significantly less infections in the NPIT group (7 versus 2; *P* = 0.024).

Gomoll *et al.* [30] reported a case series of 35 patients treated with an NPIT system following foot and ankle trauma and found no surgical site infection in NPIT-treated patients. Similarly, Reddix *et al.* [31] found no surgical site infections in a case series of 19 morbidly obese patients undergoing acetabular fracture repair followed by NPIT therapy. In a second retrospective series by the same authors [32], 66 patients undergoing acetabular repair followed by standard dressings were compared with 235 patients with acetabular repair followed by NPIT therapy. While the control group exhibited 6.1 % deep wound infections, only 1.3 % in the NPIT group showed deep wound infections.

Atkins *et al.* [33] and Colli *et al.* [27] reported a case series with a total of 67 patients using an NPIT system following cardiac surgery on sternal wounds. NPIT therapy was well tolerated and might have prevented surgical site infections in these cases.

Matatov *et al.* [34] used an NPIT system on groin wounds following vascular surgery. While surgical site infections were observed in 30 % of patients in the control group, only 6 % of wounds were infected in the NPIT group. Similar results were reported in a small single-centre trial with 50 patients (25 per group) undergoing colorectal surgery [35]. In this population, NPIT reduced infectious wound complications from 11 cases to 2 (*P* = 0.008).

A recent Cochrane review [36] evaluated NPIT therapy for skin grafts and surgical wound healing by primary intention in regard to the proportion of surgical wounds that healed completely. The review concluded that very limited evidence exists to evaluate NPIT and concluded that high-quality trials in this field are urgently needed.

Current data show that NPIT therapy can be used with few adverse events on different types of wound; however, a multicentre, high-quality randomized controlled trial evaluating NPIT therapy in abdominal surgery has not yet been performed.

### Objective

The Poniy trial aims to investigate whether the application of an NPIT device (Prevena Incision Management System, KCI, Inc., San Antonio, TX, USA) reduces the rate of surgical site infections within 30 days postoperatively, in surgical patients who underwent elective open colorectal surgery, by 50 % (from 25 % to 12.5 %). This rate of reduction is assumed based on previous studies with the NPIT device, all of which showed a reduction of at least 50 % [28, 29, 32, 34, 35]. A number of secondary outcome parameters have been defined as secondary endpoint measures to further evaluate the efficacy of the NPIT system in abdominal surgery (see section outcome measures).

## Methods/Design

### Trial sites

The Poniy trial will be performed at 15 sites of the Trial Network (CHIR-Net) of the German Surgical Society (Deutsche Gesellschaft für Chirurgie). Most of these sites have participated in previous randomized controlled trials and all centres were adequately trained and prepared according to the ICH-GCP (International Conference on Harmonisation of Technical Requirements for Registration of Pharmaceuticals for Human Use—Good Clinical Practice) rules for participation in this trial.

### Trial population and eligibility criteria

All adult (≥18 years of age) surgical patients scheduled for elective open colorectal surgery will be eligible, if they are able to understand the extent and nature of the Poniy trial and if they provide written informed consent. The following exclusion criteria were defined: (a) pregnant or breast-feeding women; (b) open abdominal surgery within the 60 days immediately prior to the trial operation; (c) planned relaparotomy within 30 days after trial operation; (d) laparoscopic or laparoscopic assisted abdominal operations; (e) patients that received preoperative antibiotic treatment.

### Sample size

A total of 152 patients will be analyzed per group. Given an estimated drop-out rate of approximately 10 %, 340 patients will be randomized to the two treatment arms.

### Type of trial

This is a randomized, controlled, observer-blinded multicentre surgical trial with two parallel study groups.

### Recruitment and trial timeline

Fifteen centres of general and abdominal surgery in Germany will participate in this trial. The centres include university hospitals and community hospitals, some of which are certified centres for colorectal surgery (Darmkrebszentrum, German Cancer Society, DKG). All centres are members of the Trial Network (CHIR-Net) of the German Surgical Society. Physicians or nurses involved in the trial have been trained in ICH-GCP prior to initiation of the trial. Informed consent will be obtained from each patient. Furthermore, all centres and participants were specifically instructed in study-specific procedures before the start of the trial. The centres will be supported by an ICH-GCP qualified flying study nurse from the CHIR-Net Surgical Regional Centre in Munich to ensure protocol conforming data acquisition and trial interventions. Stratification will be performed according to centre.

**Fig. 1:**
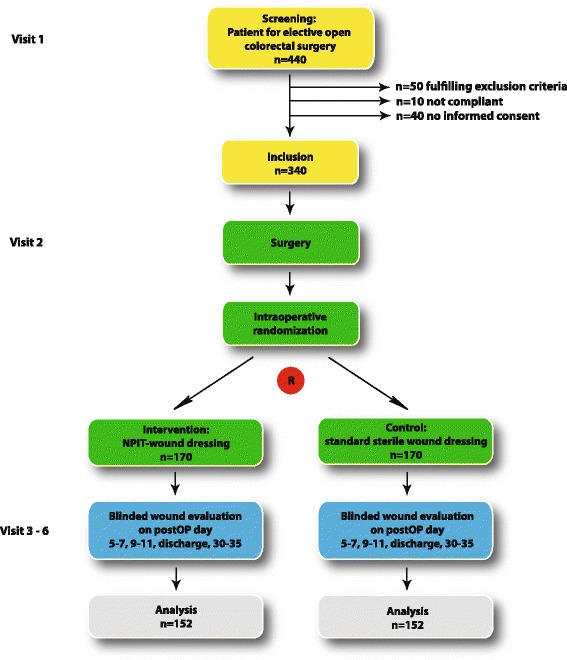
Flow chart of the Poniy trial. NPIT, negative-pressure incision therapy; postOP day, postoperative day; R, randomization

The duration of the recruitment phase is expected to be 14 months. The last follow-up will be performed a maximum of 30 days after the last patient undergoes the trial intervention. Hence, the total duration of the trial (first patient in to last patient out) is expected to be 15 months. The study flow is outlined in Fig. 1.

### Randomization and blinding

Randomization and blinding will be performed with the help of sealed, opaque, individually numbered envelopes, restricted to choosing one at a time. The envelopes contain data sheets with information regarding the group allocation and the randomization number. Both are prefabricated by a biostatistician of the *Technische Universität München* (Munich, Germany). Randomization (visit 2, see Fig. 1) will be performed in the operation theatre at the end of surgery, after closure of the skin. This prevents potential bias by different methods of wound closure or wound irrigation. An interrupted skin closure must be performed with either sutures or staplers, according to local practice.

Randomization will be stratified by centre. To assure balanced group sizes, a block-wise randomization will be applied. Basic characteristics of the patient and day of randomization must be documented on the randomization sheets. Subsequently, randomization sheets must be dated, signed and stored away from the patient records and the trial documents, as well as the investigator site file, to ensure blinding. Patients, outcome assessors and the trial statistician will be blinded for the trial intervention. The outcome assessor (postoperative surgical site infection) will therefore neither be part of the surgical team that performs the trial intervention nor take part in the postoperative care of the patient and will have no access to the randomization sheets. Blinding of patients is not feasible, as the NPIT wound dressing system is different from the standard sterile dressing. However, the primary endpoint measure (whether or not there is surgical site infection according to the CDC definition, Table 1) cannot be influenced by the subjective assessment of the patient.

**Table 1:** Definitions of abdominal surgical site infections classified according to the Centres for Disease Control and Prevention [9]

Superficial incisional surgical site infections	Deep incisional surgical site infections^a^	Organ or space surgical site infections
1. Infection occurs within 30 days after the operation, and	1. Infection occurs within 30 days after the operation, and	1. Infection occurs within 30 after the operation, and
2. Infection involves only skin or subcutaneous tissue of the incision, and	2. Infection involves deep soft tissues (e.g., fascial and muscle layers) of the incision, and	2. Infection involves any part of the anatomy (e.g., organs or spaces), other than the incision, which was opened or manipulated during an operation, and
3. At least one of items A to D	3. At least one of items A to D	3. At least one of items A to D
A. Purulent drainage, with or without laboratory confirmation, from the superficial incision	A. Purulent drainage from the deep incision but not from the organ or space component of the surgical site	A. Purulent drainage from a drain that is placed through a stab wound^b^ into the organ or space
B. Organisms isolated from an aseptically obtained culture of fluid or tissue from the superficial incision	B. A deep incision spontaneously dehisces or is deliberately opened by a surgeon when the patient has at least one of the following signs or symptoms: fever (>38 °C), localized pain, or tenderness, unless site is culture-negative	B. Organisms isolated from an aseptically obtained culture of fluid or tissue in the organ or space
C. At least one of the following signs or symptoms of infection: pain or tenderness, localized swelling, redness, or heat and superficial incision is deliberately opened by surgeon, unless incision is culture-negative	C. An abscess or other evidence of infection involving the deep incision is found on direct examination, during reoperation, or by histopathologic or radiological examination	C. An abscess or other evidence of infection involving the organ or space that is found on direct examination, during reoperation, or by histopathologic or radiological examination
D. Diagnosis of superficial incisional surgical site infections by the surgeon or attending physician	D. Diagnosis of a deep incisional surgical site infection by a surgeon or attending physician	D. Diagnosis of an organ or space surgical site infection by a surgeon or attending physician

^a^Report infection that involves both superficial and deep incision sites as deep incisional surgical site infection; report organ or space surgical site infection that drains through the incision as deep incisional surgical site infection

^b^If the area around a stab wound becomes infected, it is not a surgical site infection; it is considered a skin or soft tissue infection, depending on its depth

### Interventions

The schedule of trial interventions is presented in Table 2.

**Table 2:** Study visits of the Poniy trial

Activity	Visit 1 (screening, inclusion)	Visit 2 (operation + randomization)	Visit 3 (postoperative day 5–7)	Visit 4 (postoperative day 9–11)	Visit 5 (discharge)	Visit 6 (postoperative day 30–35)	Optional visit (anytime between visits 2 and 6)
Inclusion and exclusion criteria	×						
Informed consent	×						
Medical history	×						
Physical examination	×						
Surgery		×					
Randomization		×					
Documentation of surgical site infection^a^			×^b^	×^b^	×^b^	×^b^	×^b^
Documentation of other wound complications^c^			×				×
Measurement of wound length			×				
Documentation of wound pain (visual analogue scale, 1–10)			×	×	×	×	×
Documentation of reoperation			×	×	×	×	×
Documentation of adverse or serious adverse events		×	×	×	×	×	×
Documentation of antibiotic-therapy			×	×	×	×	×

^a^By blinded wound assessor according to definition of US Centers for Disease Control and Prevention

^b^In case of surgical site infection, a microbiological swab according to local practice should be obtained for microbiological specification and antimicrobial testing

^c^Documented as ‘yes’ if blister formation and ‘no’ otherwise

#### Experimental intervention

**Fig. 2:**
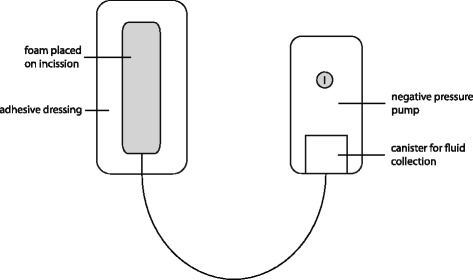
Negative-pressure incision therapy wound device used in the Poniy trial. The sterile foam is placed on the skin incision immediately after skin closure and attached to the skin via the adhesive dressing. A fixed negative pressure of −75 mmHg to −125 mmHg is applied via the negative-pressure pump and the attached tubing. Wound secretions are collected in a canister, which is integrated in the pump

Patients undergoing open colorectal procedures and randomized to the experimental arm will have the full length of their incision covered with an NPIT device (Prevena Incision Management System, KCI, Inc., San Antonio, TX, USA) starting in the operating room right after closure of the skin incision (see Fig. 2). In the intervention group, the NPIT system should cover the surgical wound for a minimum of 5 days and a maximum of 7 days (till postoperative day 5–7; visit 2; see Table 2). If the NPIT system is removed prior to postoperative day 5–7 (event visit; see Table 2) the reasons must be documented and a new NPIT system has to be applied to ensure coverage of the wound for 5–7 days after the operation. The reasons for a definite removal of the NPIT system prior to postoperative day 5–7 must be documented and state whether there is a surgical site infection (the primary endpoint measure of the trial) or whether the NPIT system is not tolerated by the patient.

#### Control intervention

Incisions will be covered with standard sterile dressings postoperatively. Frequency of dressing changes depends on local practice.

### Risks

No additional risks for study patients are anticipated, since the safety and feasibility of the application of the NPIT device on postoperative abdominal wounds has been established in several previous studies. The NPIT device used in Poniy trial is CE (Conformité Européenne) certified. The surgical procedures carried out within Poniy are not affected by the trial.

### Outcome measures

The primary efficacy endpoint measure of the Poniy trial is the rate of surgical site infections within 30 days after the operation, according to CDC criteria, which constitute an internationally accepted standard definition [9] (Table 1). If the patient’s wound cannot be evaluated on postoperative day 30, a clinical evaluation according to the CDC criteria up to postoperative day 35 will be allowed.

The following outcome measures have been defined as secondary endpoint measures:

- Duration of hospital stay (in days);
- Rate of reoperation in both groups;
- Duration of postoperative antibiotic treatment in both groups within 30 days (in days);
- Duration of NPIT therapy (in days) in the interventional arm;
- Wound pain, as assessed by a visual analogue scale (graded from 1 to 10) in both groups;
- Rate of wound complications other than wound infections in both groups;
- Number of postoperative severe adverse events in both groups within 30 days.

### Data management

All required information collected during the trial will be entered in a case record form by the investigator or a designated representative. Documentation is expected to be completed as soon as possible after information has been collected. The investigator is responsible for the accuracy of the documentation and must ensure that all entries can be verified by source data. An explanation must be given for all missing data. Corrections in the case record form must be signed, dated and leave the corrected entry visible. The completed case record form must be reviewed, signed and dated by the investigator named in the trial protocol or by a designated sub-investigator. After a copy is made for retention at the trial centre, the original case record form is sent in a sealed envelope by certified mail to the Centre for Data Management at the Münchner Studienzentrum, a member of the Network of Coordinating Centres for Clinical Trials (Koordinierungszentren für Klinische Studien Network) at the Technische Universität München, Munich, Germany. Double data entry is performed by data management according to standard operating procedures predefined in the data management plan to ensure correct transfer of data from the case record form to the database (MACRO™ version 3, Microsoft SQL Datenbank, using Microsoft Internet Explorer version 6 or higher). Completeness, validity and plausibility of data are examined by validating programs as well as individual inspection and any queries generated as a consequence must be clarified by the investigator or designated sub-investigator. At the end of the trial, the principal investigator will retain the original case record forms.

### Monitoring

Monitoring of the trial data will be performed by an independent institution experienced in the monitoring of surgical trials (Koordinierungszentren für Klinische Studien Network) at the Münchner Studienzentrum. Monitoring will be carried out in accordance with ICH-GCP guidelines [37] and standard operating procedures of the Münchner Studienzentrum, to ensure patients’ safety and integrity of the clinical data, e.g., primary outcome measure in adherence to study protocol. All trial sites are activated with an initiation visit by the monitor or CHIR-Net coordinator, who will deliver and explain the investigator site file, discuss relevant issues and train trial personnel in study-specific interventions. Regular contact by phone or email with all participating centres will enable the CHIR-Net coordinator and the monitor to control study progression and adherence to the study protocol, and to discuss problems related to the study. Regular on-site monitoring visits are planned for all sites. Investigators must allow the monitor to look at all essential documents, support the monitor during visits and answer queries. All monitoring procedures will be predefined in a trial-specific monitoring manual. In addition, a GCP-trained flying study nurse employed by the CHIR-Net regional centre Munich will assist the trial sites with documentation and data collection if needed. Furthermore, close-out visits are planned for each centre.

### Safety evaluation and reporting of adverse events

An adverse event is defined as any untoward medical occurrence in a patient that does not necessarily have a causal relationship with the trial treatment and that occurs between inclusion of the patient and visit 6. The following exceptions are predefined in the study protocol and will not be recorded as adverse events: (1) occurrence of surgical site infection (the primary endpoint measure) is assessed as endpoint measure only (not as an adverse event); (2) any adverse event that is expected during the postoperative course or the underlying disease (e.g., pain, nausea, vomiting, hypertension, hypotension, imbalances of blood sugar or electrolytes or other laboratory values out of range) and that does not exceed grade I of the Dindo–Clavien classification of postoperative complications [38, 39] (Table 3). Assessment will be performed by the investigator or the designated sub-investigator.

**Table 3:** Dindo–Clavien definition of postoperative complications [38]

Grade	Definition
I	Any deviation from the normal postoperative course without the need for pharmacological treatment or surgical, endoscopic or radiological intervention
	Allowed therapeutic regimens are: drugs as antiemetics, antipyretics, analgesics, diuretics, electrolytes and physiotherapy
	This grade also includes wound infections opened at the bedside
II	Requiring pharmacological treatment with drugs other than those allowed for grade I complications
	Blood transfusions and total parenteral nutrition are also included
III	Requiring surgical, endoscopic or radiological intervention
IIIa	Intervention, not under general anesthesia
IIIb	Intervention, under general anesthesia
IV	Life-threatening complication (including central nervous system complications)^a^ requiring intermediate care or intensive care unit management
IVa	Single organ dysfunction (including dialysis)
IVb	Multiorgan dysfunction
V	Death of a patient
Suffix ‘d’	If the patient suffers from a complication at the time of discharge (see examples in Table 1), the suffix ‘d’ (for ‘disability’) is added to the respective grade of complication. This label indicates the need for a follow-up to fully evaluate the complication.

^a^Brain haemorrhage, ischaemic stroke, subarachnoidal bleeding, but excluding transient ischaemic attacks

From the day the patient signs informed consent until the regular end of the trial (visit 6) or until premature withdrawal of the patient, all serious adverse events will be documented on a serious adverse event form, available in the investigator site file. A serious adverse event will be defined as an event that results in death, is immediately life-threatening, requires or prolongs hospitalization, or results in persistent or clinically important disability or incapacity, as judged by the investigator or designated sub-investigator. Serious adverse events will be classified by intensity (mild, moderate, severe), outcome (ongoing, recovered completely, recovered with sequelae, death, unknown) and causality (unrelated; possibly, probably or definitely related to trial intervention; not assessable). The assessment is based on clinical findings and needs to be made by the investigator or designated sub-investigator in the participating trial centre. Serious adverse events must be reported within 7 days after becoming known.

### Statistical methods

#### Sample size calculation

Sample size calculation is based on the primary endpoint measure (whether or not there is surgical site infection, according to CDC) within 30 days post operation [9] and was conducted by using nQuery Advisor® software version 7.0 (Statistical Solutions Ltd, Cork, Ireland). Based on the assumption that the percentage of patients developing postoperative wound infections in a surgical population undergoing open colorectal surgery is approximately 25 % for the control group (based on results from previous randomized controlled trials in abdominal surgery [14, 15, 17]) and can be reduced to 12.5 % in the experimental intervention arm, a group sample size of 152 patients would need to be compared using the chi-square test, to achieve 80 % power in detecting this difference in surgical site infection rate at a two-sided level of significance of 5 %. Under the assumption of a drop-out rate of up to 15 %, a total of 340 patients (170 per group) need to be enrolled in the study. Owing to the broad inclusion criteria and the limited number of exclusion criteria, the limited study time per patient (30 days), as well as the comprehensible nature of the trial, no more than 100 patients are expected to fail the screening process. Therefore, the total number of patients needed for screening is 440 (Fig. 1).

#### Analysis populations

##### Intention-to-treat population

The intention-to-treat population contains all patients who have participated in visit 2 (surgery), independent of the intervention they receive (control or NPIT). Analysis will occur as randomized. Patients who unexpectedly receive a laparoscopic or laparoscopic assisted operation (exclusion criterion) will be excluded from the intention-to-treat population. Missing primary endpoint data in the intention-to-treat population will be treated as follows: data missing because of death or relaparotomy will be counted as surgical site infection in both groups. Data missing for other reasons (e.g., lost to follow-up) will not be counted as surgical site infection in either group.

##### Per-protocol population

The per-protocol population contains all patients included in the intention-to-treat population as treated (not as randomized). The following patients will be excluded: (a) patients who receive a surgical wound dressing that is not predefined in the randomization scheme (i.e., neither an NPIT system nor a standard sterile dressing); (b) patients who do not reach visit 6 for reasons other than death or relaparotomy. Patients who die or undergo relaparotomy will be counted as having surgical site infections in both groups.

##### Surgical site infection population

The surgical site infection population contains all patients included in the intention-to-treat population who have reached the study end (visit 6), independent of the treatment they have received (‘as randomized’).

##### Safety population

The safety population contains all patients who have started visit 2 independent of the treatment they have received (analysis ‘as randomized’).

#### Analysis of the primary endpoint measure

The analysis of the primary endpoint measure will be performed in the intention-to-treat population. The statistical hypothesis is:

$$ {H}_0:{\varPi}_{\mathrm{T}}={\varPi}_{\mathrm{C}}\kern1em \mathrm{versus}\kern1em {H}_{\mathrm{A}}:{\varPi}_{\mathrm{T}}\ne {\varPi}_{\mathrm{C}} $$

where Π_T_ is the rate of surgical site infections in the NPIT group and Π_C_ the rate of surgical site infections in the control group. Rates of surgical site infection will be analyzed via multivariate binary logistic regression with centre effects and possible baseline differences between intervention and control group. The level of significance will be set at 5 %.

#### Sensitivity analysis of the primary endpoint measure

The primary endpoint analysis will be repeated in the per-protocol and surgical site infection population. Further sensitivity analyses will be performed as follows: (a) in the surgical site infection population, all missing data will be imputed as surgical site infections in both groups; (b) in the surgical site infection population, all missing data will be imputed as non-surgical site infection in both groups; (c) in the surgical site infection population, missing data will be imputed as surgical site infections in the intervention group, but as non-surgical site infection in the control group; (d) a time-to-event analysis will be performed in the surgical site infection population according to Kaplan–Meier analysis.

#### Prespecified subgroup analysis

Prespecified subgroup analysis will be performed in the surgical site infection population for the depth of surgical site infection (superficial versus deep versus organ space; Table 1) in both groups (NPIT versus control). Furthermore, the rate of all surgical site infections will be analyzed in the surgical site infection population in the following subgroups: (a) National Nosocomial Infection Surveillance risk score (0 versus 1 versus >1); (b) body mass index (<25 versus >25); (c) grade of contamination (clean versus clean-contaminated versus contaminated versus dirty, as defined by the CDC, [9]); (d) American Society of Anesthesiology score (1 and 2 versus 3 versus ≥4); (e) presence of an ostomy (yes versus no); (f) skin preparation used (ethanol-based versus isopropyl-alcohol-based versus chlorhexidine-based versus Povidone-iodine-based); (g) age (≤65 versus > 65 years), (g) cause of surgery (malignant versus benign surgery).

#### Analysis of secondary endpoint measures

All secondary endpoints will be analyzed using descriptive statistical methods and comparisons between groups will be performed with the appropriate statistical tests. For comparisons of frequencies between groups, the chi-square test and, if appropriate, the Fisher exact test, will be used. As appropriate, Student’s *t* test, the Mann–Whitney U test or analysis of covariance (ANCOVA) will be employed for group comparisons of quantitative data. All tests will be two-sided at a significance level of 5 %.

#### Safety analysis

For safety analysis, all adverse and serious adverse events will be analyzed via descriptive statistical methods. For comparisons of frequencies between groups, the chi-square test and, if appropriate, the Fisher exact test, will be used. Patients who have started visit 2 (operation) will be analyzed as treated.

Procedures for the statistical analysis of the primary and secondary endpoint measures will be conducted in line with the GCP-ICH E9 guideline [40]. For the statistical analysis, IBM SPSS Statistics version 21.0 will be used (IBM Corp., Armonk, NY). Statistical analysis will be performed by a group-allocation blinded statistician from the Institute for Medical Statistics and Epidemiology of the Technische Universität München.

### Withdrawals

Patients are free to withdraw from trial participation at their own request at any time and without giving reasons for their decisions. Withdrawals will be documented in the case record form and in the patient’s medical record. Furthermore, all ongoing serious adverse events must be followed up and documented until their final outcome can be determined.

### Stopping guidelines

The trial can be prematurely closed by the coordinating investigator in consultation with the responsible biostatistician for the following reasons:

- It appears that patients’ enrolment is unsatisfactory with respect to quality or quantity, or data recording is severely inaccurate or incomplete.
- There is external evidence demanding a termination of the trial, e.g., indicating that the rate or severity of serious adverse events or morbidity in this trial poses a potential health hazard caused by the trial treatment in one or both of the trial groups.

In case of premature closure, the ethics committee must be informed.

### Trial organization and administration

#### Funding

The NPIT devices in the intervention group (Prevena Incision Management System) are provided by KCI Inc., Wiesbaden, Germany. Furthermore, financial support to cover costs for data management, monitoring and trial coordination are provided by KCI Inc. (San Antonio, TX, USA). There are no restrictions on publications and no conflict of interest. The idea for the Poniy trial was conceived, the trial protocol written and the trial initiated independently of any industrial funder. Industrial funders and trial management are independent.

#### Ethical approval

Before the start of the trial, the trial protocol, informed consent document and any other trial documents were approved by the ethics committee of the *Klinikum rechts der Isar*, Technische Universität München, Munich, Germany on 19 May 2014 (number 155/14). The trial protocol, informed consent and trial documents must be approved by the respective ethics committees of all participating centres. Recruitment will not begin in any individual centre until all local approvals have been obtained.

#### Registration

The trial protocol is registered at the German Clinical Trials Register (part of the World Health Organization International Clinical Trials Registry Platform), number DRKS00006199.

#### Good clinical practice

The procedures set out in this trial protocol, pertaining to the conduct, evaluation and documentation of this trial, are designed to ensure that all persons involved in the trial abide by GCP [37] and the ethical principles described in the current revision of the Declaration of Helsinki [41]. The trial will be carried out in keeping with local legal and regulatory requirements.

## Discussion

Postoperative surgical site infections are amongst the most frequent surgical complications, affecting approximately 14 % to 32 % of patients undergoing abdominal surgery [18, 42, 43]. These numbers have changed little over the last 20 years despite internationally accepted recommendations for control of surgical site infections (reviewed in [44]). In prospective trials with clear definitions, surgical site infection rates above 15 % have frequently been reported [13–16, 18] and are especially high in colorectal surgery [14, 15, 17]. Applying standard definitions for surgical site infections is crucial, as a number of surgical site infections occur after discharge of patients from the hospital and would thus remain unnoticed if standardized wound evaluation, as well as adequate follow-up, were not applied. Unfortunately, numerous different surgical site infection definitions have been proposed [45], albeit the surgical site infections definition of the CDC has gained wide acceptance over past decades [9]. According to this definition, surgical site infections are grouped into superficial, deep and organ-space surgical site infections that occur within 30 days of the operation (Table 1).

The most frequent pathogens causing postoperative surgical site infections in general surgical patients are *Staphylococcus aureus*, *Escherichia coli* and *Enterococcus* species. Similarly, in abdominal surgical patients, *E. coli*, *Enterococcus* species, *Enterobacter* species and *S. aureus* are the most frequently pathogens isolated from wounds [7]. These data indicate that endogenous contaminations from the patients’ skin or the gastrointestinal tract occurring during surgery account for most surgical site infections. Therefore, a considerable number of surgical site infections might be prevented by draining contaminated wound secretions from the incisional site during the early postoperative period. Negative-pressure incision therapy devices (Fig. 2) have been designed to drain wound secretions from the postoperative wound. The feasibility and safety of NPIT devices has been demonstrated in several studies [28–32, 34], but their efficacy has not yet been tested in multicentre randomized controlled trials. However, previous studies were single-centre trials, lacked clear surgical site infection definitions and endpoint measures, or included only few patients [36]. Furthermore, no data exists on the efficacy of NPIT devices in abdominal surgery.

The Poniy trial was designed to test the efficacy of NPIT in comparison with standard sterile dressings in a group at high risk of surgical site infection, i.e., patients undergoing open colorectal surgery. In similar patient cohorts, surgical site infection rates in randomized controlled trials using the CDC definition have consistently been reported as above 20 % [14, 15, 17, 18]. Since further single-centre studies would not increase the external validity (generalizability), a multicentre approach was chosen and the trial was initiated within the Trial Network of the German Surgical Society (CHIR-Net). To further increase external validity, broad inclusion criteria and only a few exclusion criteria will be applied, allowing for the screening and recruitment of a large number of elective colorectal surgical cases in participating hospitals. Hospitals of different care levels will participate in this trial together, underlining the pragmatic approach of the trial. For many participating surgical departments, surgical site infections represent the most frequent postoperative complication and thus a pressing surgical problem that remains to be solved. To ensure data quality, members of all participating centres are trained in GCP guidelines, trial intervention, documentation and blinding. In addition, internal validity is ensured by patient- and observer-blinding, application of definite endpoint measures (surgical site infection definition by the CDC) and complete outcome reporting and follow-up for 30 days. Applying high methodological standards, the results of the trial should help to improve surgical treatment of patients.

## Trial status

Planned recruitment will start on 1 October 2015.

## Abbreviations

ANCOVA: analysis of covariance
CDC: US Centers for Disease Control and Prevention
CE: Conformité Européenne
CHIR-Net: Trial Network of the German Surgical Society
GCP: Good Clinical Practice
ICH: International Conference on Harmonisation of Technical Requirements for Registration of Pharmaceuticals for Human Use
NPIT: negative-pressure incision therapy
